# A Mobile Clinical Decision Support System for High-Risk Pregnant Women in Rural India (SMARThealth Pregnancy): Pilot Cluster Randomized Controlled Trial

**DOI:** 10.2196/44362

**Published:** 2023-07-20

**Authors:** Shobhana Nagraj, Stephen Kennedy, Vivekananda Jha, Robyn Norton, Lisa Hinton, Laurent Billot, Eldho Rajan, Ameer Mohammed Abdul, Anita Phalswal, Varun Arora, Devarsetty Praveen, Jane Hirst

**Affiliations:** 1 Nuffield Department of Women's & Reproductive Health University of Oxford Oxford United Kingdom; 2 The George Institute for Global Health Imperial College London London United Kingdom; 3 Health Systems Collaborative Nuffield Department of Medicine University of Oxford Oxford United Kingdom; 4 Oxford University Global Surgery Group University of Oxford Oxford United Kingdom; 5 The George Institute for Global Health New Delhi India; 6 University of New South Wales Sydney Australia; 7 Prasanna School of Public Health Manipal Academy of Higher Education Manipal India; 8 The George Institute for Global Health UNSW Sydney Kensington Australia; 9 Nuffield Department of Primary Care Health Sciences University of Oxford Oxford United Kingdom; 10 Post Graduate Institute of Medical Science Rohtak India

**Keywords:** decision support systems, clinical, telemedicine, community health workers, pregnancy, high risk, diabetes, gestational, cardiovascular diseases

## Abstract

**Background:**

Cardiovascular disease (CVD) is the leading cause of death in women in India. Early identification is crucial to reducing deaths. Hypertensive disorders of pregnancy (HDP) and gestational diabetes mellitus (GDM) carry independent risks for future CVD, and antenatal care is a window to screen and counsel high-risk women. In rural India, community health workers (CHWs) deliver antenatal and postnatal care. We developed a complex intervention (SMARThealth Pregnancy) involving mobile clinical decision support for CHWs and evaluated it in a pilot cluster randomized controlled trial (cRCT).

**Objective:**

The aim of the study is to co-design a theory-informed intervention for CHWs to screen, refer, and counsel pregnant women at high risk of future CVD in rural India and evaluate its feasibility and acceptability.

**Methods:**

In phase 1, we used qualitative methods to explore community priorities for high-risk pregnant women in rural areas of 2 diverse states in India. In phase 2, informed by behavior change theory and human-centered design, we used these qualitative data to develop the intervention components and implementation strategies for SMARThealth Pregnancy in an iterative process with end users. In phase 3, using mixed methods, we evaluated the intervention in a cRCT with an embedded qualitative substudy across 4 primary health centres: 2 in Jhajjar district, Haryana, and 2 in Guntur district, Andhra Pradesh.

**Results:**

SMARThealth Pregnancy embedded a total of 15 behavior change techniques and included (1) community awareness programs; (2) targeted training, including point-of-care blood pressure and hemoglobin measurement; and (3) mobile clinical decision support for CHWs to screen women in their homes. The intervention focused on 3 priority conditions: anemia, HDP, and GDM. The evaluation involved a total of 200 pregnant women, equally randomized to intervention or enhanced standard care (control). Recruitment was completed within 5 months, with minimal loss to follow-up (4/200, 2%) at 6 weeks postpartum. A total of 4 primary care doctors and 54 CHWs in the intervention clusters took part in the study. Fidelity to intervention practices was 100% prepandemic. Over half the study population was affected by moderate to severe anemia at baseline. The prevalence of HDP (2.5%) and GDM (2%) was low in our study population. Results suggest a possible improvement in mean hemoglobin (anemia) in the intervention group, although an adequately powered trial is needed. The model of home-based care was feasible and acceptable for pregnant or postpartum women and CHWs, who perceived improvements in quality of care, self-efficacy, and professional recognition.

**Conclusions:**

SMARThealth Pregnancy is an innovative model of home-based care for high-risk pregnant women during the transitions between antenatal and postnatal care and adult health services. The use of theory and co-design during intervention development facilitated acceptability of the intervention and implementation strategies. Our experience has informed the decision to initiate a larger-scale cRCT.

**Trial Registration:**

ClinicalTrials.gov NCT03968952; https://clinicaltrials.gov/ct2/show/NCT03968952

**International Registered Report Identifier (IRRID):**

RR2-10.3389/fgwh.2021.620759

## Introduction

India has witnessed an epidemiological transition to noncommunicable diseases (NCDs) over the last 3 decades [1]. Cardiovascular disease (CVD) is the leading cause of death in women in India [2], and rural women are particularly vulnerable due to limited health literacy and health care access. Pregnancy-related conditions including hypertensive disorders of pregnancy (HDP) and gestational diabetes mellitus (GDM) are independent risk factors for future cardiometabolic disorders (CMDs), including type 2 diabetes mellitus and CVD [3-7]. Pregnancy is a time of increased receptivity of women to behavior change [8]. Early identification of at-risk pregnant women, preventative measures, and counseling about the likelihood of future cardiometabolic complications may help slow progression to CVD and reduce mortality.

Several reviews have demonstrated the role of mobile health (mHealth) technologies in improving maternal and neonatal health services [8,9]. mHealth interventions have the potential to improve equity and access to timely, affordable antenatal care (ANC) [10-12]; support self-management in relation to GDM [13] and gestational weight gain [14]; and support women in low-resource settings [15]. Benefits to perinatal care extend to both patients and clinicians [8]; however, there is limited evidence of impact on health outcomes [9], cost-effectiveness, and implementation within rural settings [8].

In India, community health workers (CHWs), known as accredited social health activists (ASHAs) and auxiliary nurse midwives (ANMs), deliver ANC and postnatal care (PNC) to women living in rural villages. ASHAs are educated to a secondary school level and serve a population of approximately 1000 people. They visit pregnant women in their homes and work closely with ANMs in subcenters to refer high-risk cases to the nearest primary care center (staffed by a primary care doctor) or secondary care facility (for specialist support), for which there are significant workforce shortages [16]. Task sharing with CHWs using mHealth technology to provide clinical decision support in rural India has been shown to be feasible for detection, referral, and management of NCDs in nonpregnant adults [17,18] and for HDP [19-22]. There is, however, a paucity of high-quality evidence to guide postnatal management in low-resource settings of women following a high-risk pregnancy, including those with multimorbidity. Innovative solutions are required to address the needs of rural women, particularly during the transitions between ANC and PNC and adult health services.

We, therefore, developed a complex intervention, SMARThealth Pregnancy, building upon the Systematic Medical Appraisal Referral and Treatment in India (SMARThealth India) program [18], to improve care for rural pregnant women with high-risk conditions that placed them and their baby at immediate risk during pregnancy and at future risk of CMDs. Our objectives were to (1) co-design a theory-informed complex intervention and implementation strategies for CHWs to screen, refer, and counsel pregnant women in their homes using established clinical guidelines and (2) evaluate the feasibility and acceptability of implementation and preliminary effectiveness of the intervention for CHWs and pregnant women. We worked across 2 diverse districts of rural India—the Jhajjar district, Haryana, and the Guntur district, Andhra Pradesh.

## Methods

### Ethics Approval

Ethics approvals for the study were obtained from the Oxford Tropical Research Ethics Committee (OxTREC references: 501-19 [usability study] and 22-19 [pilot study]) and the George Institute India Ethics Committee (references: 03/2019 and 010/2019), with trial registration (ClinicalTrials.gov NCT03968952) and oversight by a steering committee. Participant information sheets were shared both verbally and in written format in local languages, and written informed consent was obtained from all participants.

### Development of the SMARThealth Pregnancy Intervention

The design and development of SMARThealth Pregnancy occurred in 3 phases, guided by the methodological principles of human-centered design (inspiration, ideation, and implementation) [23,24]. First, we conducted in-depth contextual work at both study sites to explore local priorities for care, the sociocultural practices around childbirth and the postpartum period, and the components required for a complex intervention [16]. This previously published qualitative study highlighted 3 key priority areas identified by the communities in relation to high-risk pregnant women (anemia, HDP, and GDM).

**Figure 1:**
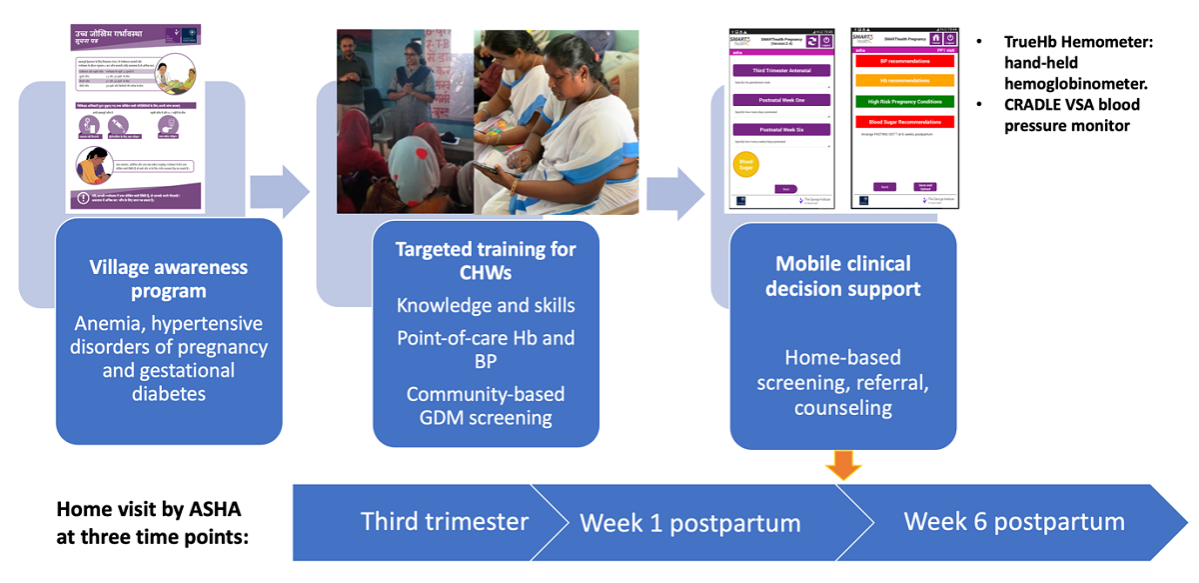
SMARThealth Pregnancy intervention components. ASHA: accredited social health activist; BP: blood pressure; CHW: community health worker; GDM: gestational diabetes mellitus; Hb: hemoglobin.

Second, the capability, opportunity, and motivation model of behavior change (COM-B)/behavior change wheel framework [25] was used, which outlines 3 interacting domains that affect behavior change: capability refers to a physical and psychological capacity to engage in an activity; opportunity refers to the physical and social factors that influence behavior change; and motivation refers to both automatic impulses and reflective processes that direct behavior. We mapped qualitative data arising from early contextual work [16] to identify barriers to the capabilities, opportunities, and motivations of CHWs and pregnant women in relation to improving care for anemia, HDP, and GDM [26]. We then selected intervention functions and behavior change techniques [27] most likely to be effective in overcoming targeted barriers and leading to improved care for high-risk pregnant women. Behavior change techniques are the “active ingredients” of behavior change interventions and are outlined in an extensive taxonomy [27]. Intervention functions include education, persuasion, incentivization, coercion, training, restriction, environmental restructuring, modeling, and enablement [25]. For example, CHWs and pregnant women lacked knowledge (psychological capability) of the serious consequences of anemia in pregnancy. We overcame this barrier through (1) an awareness program on high-risk pregnancies (education), (2) targeted education and training of CHWs (training), and (3) provision of point-of-care testing and clinical decision support for CHWs to refer and counsel women at home (enablement). Figure 1 presents an overview of the final intervention components.

### Development of Mobile Clinical Decision Support System

Plain language rules were developed using established, evidence-based, country-specific clinical guidelines [28-32]. Guideline flowcharts were used to determine (1) input variables, for example, patient ID, date of birth, weight, height, last menstrual period, and hemoglobin (Hb); (2) calculated variables, for example, age, BMI, and estimated date of delivery; and (3) output recommendations, for example, “This woman has evidence of severe hypertension. Call ambulance and refer patient to hospital urgently.” Clinical and statistical validation of the algorithms was conducted in accordance with the George Institute for Global Health’s established protocol for SMARThealth platforms, using a 2-step process [33]. In step 1 (clinical validation), a deidentified data set of 200 pregnant women’s blood pressure (BP) values and pulse and Hb values, covering a range of clinical scenarios, was created. An independent senior clinician made expert recommendations based on the clinical guideline algorithms in all 200 cases. There was a 100% fit between the clinician and the rules engine outputs for the data set. Step 2 (statistical validation) involved comparison with an independently coded, randomly generated data set of 10,000 patients to include a wide range of case scenarios for all the input variables contained within the algorithms using SAS (version 9.4; SAS Institute). Outputs generated from the clinical decision support system and SAS were compared in an iterative process until there was a 100% match between the expected and actual output data.

**Figure 2:**
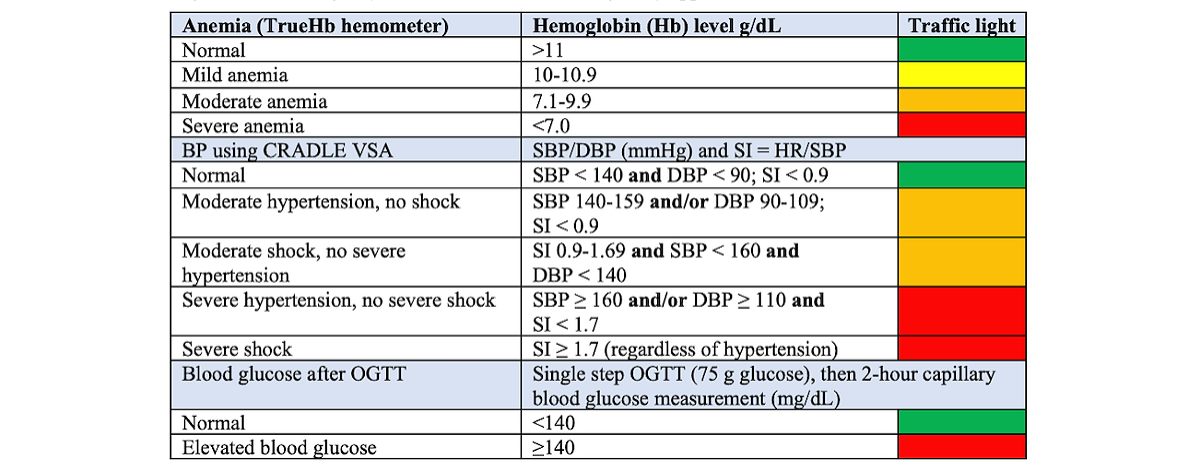
Traffic light system on SMARThealth Pregnancy app with values. BP: blood pressure; DBP: diastolic blood pressure; HR: heart rate; OGTT: oral glucose tolerance test; SBP: systolic blood pressure; SI: Shock Index.

Clinical decision support involved a 4-step process: patient registration, past obstetric and medical history, BP and Hb measurements, results of oral glucose tolerance test (OGTT) for GDM, and referral and counseling advice. A traffic light system was used to highlight readings that were of concern requiring immediate referral and management (red), moderate concern requiring referral (amber or yellow), or normal values (green) and to highlight missing ANC practices (Figure 2).

**Figure 3:**
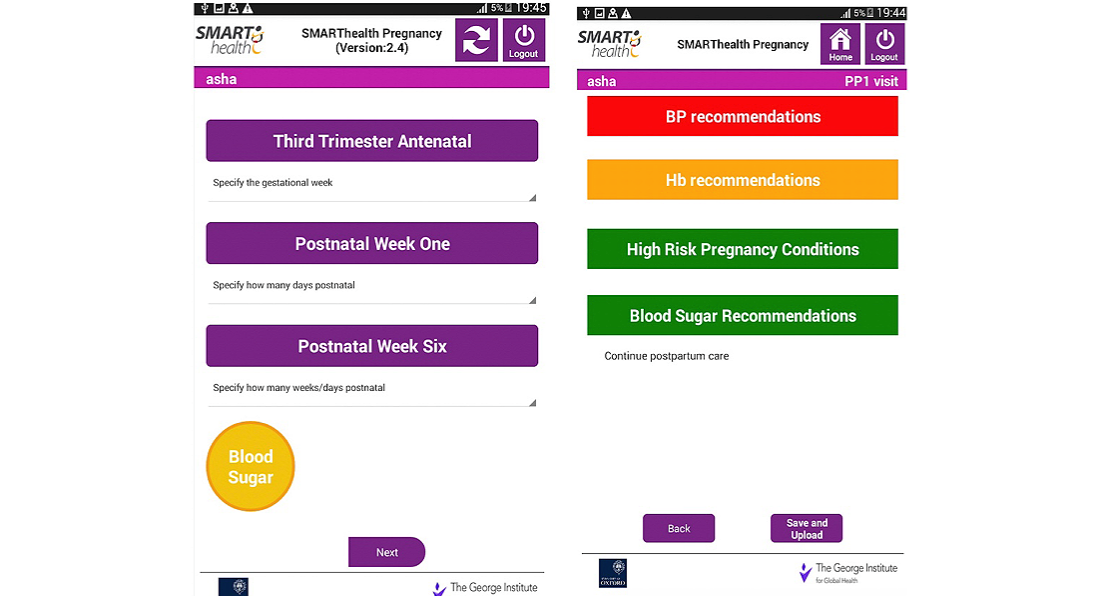
SMARThealth Pregnancy mHealth platform screens using 3 visits and a traffic light system to guide subsequent referral and counseling for community health workers. BP: blood pressure; Hb: hemoglobin.

We conducted 4 rounds of iterative usability testing with end users (CHWs and primary care doctors) using “mock” cases at both study districts to determine visit completion times, “clicks” to completion, and “think aloud” feedback and discussions, and we refined the prototype app before evaluation [26]. The front-end screens are illustrated in Figure 3.

The mHealth platform used the Android operating system, was delivered through a 7-inch mobile tablet, and was available in English, Hindi, and Telugu languages. The app used minimal data (kilobytes), worked offline, and synchronized with low-band connectivity. Data entered by CHWs were confidentially uploaded to a patient record using Open MRS software (Open-MRS) [34] to a secure server based at the George Institute for Global Health, India, which could synchronize to a sister tablet held by the primary care doctor at the local primary health center (PHC).

### Evaluation of SMARThealth Pregnancy

Guided by the Medical Research Fellowship framework for complex interventions [35], we conducted a prospective, parallel, unblinded cluster randomized controlled trial (cRCT) with 1:1 allocation in Jhajjar, Haryana, and Guntur, Andhra Pradesh (ClinicalTrials.gov NCT03968952) [36] (see CONSORT checklist in Multimedia Appendix 1). Clusters were defined as PHCs, including all affiliated health professionals. The full study protocol outlining the sample size and inclusion and exclusion criteria was developed using the CONSORT 2010 statement extension for randomized pilot and feasibility trials and has been previously published [37]. The study took place between October 2019 and December 2020 and involved 4 PHC clusters: 2 in Jhajjar district, Haryana, and 2 in Guntur district, Andhra Pradesh. The aims of the study were to determine the feasibility and acceptability of the SMARThealth Pregnancy intervention and identify any barriers to implementation in a real-world setting ahead of a larger clinical trial.

### Randomization, Recruitment, and Consent

PHCs meeting the inclusion criteria in the 2 study districts were stratified by geographical location and population size. A random number generator was used by an independent, blinded statistician (LB) to randomize 1 PHC in each state to the SMARThealth Pregnancy intervention and 1 to the control group (enhanced standard care). Written informed consent was obtained at the cluster level from the PHC administrative lead prior to randomization.

Eligible participants in the last trimester of pregnancy (28 and 36 weeks gestation) were identified by CHWs and recruited with written informed consent in their local language by a member of the study team. Women in the intervention group consented to sharing their antenatal records and having their BP and Hb measured by an ASHA at each of the 3 study visits. Women in the control group consented to share their ANC record and have BP and Hb measured by an independent team member at baseline and at 6 weeks postpartum (end-line visit). For the qualitative substudy, individual informed consent was obtained from CHWs and pregnant or postpartum women prior to interviews and focus group discussions (FGDs).

### Intervention Group Practices

**Figure 4:**
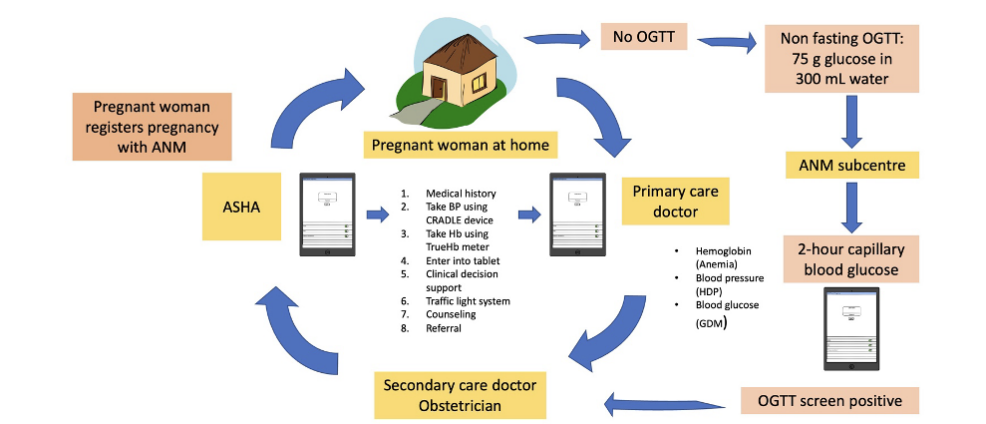
SMARThealth Pregnancy intervention practices. ANM: auxiliary nurse midwife; ASHA: accredited social health activist; BP: blood pressure; GDM: gestational diabetes mellitus; Hb: hemoglobin; HDP: hypertensive disorders of pregnancy; OGTT: oral glucose tolerance test.

CHWs in the intervention group underwent targeted training delivered over five 3-hour sessions (2.5 days), followed by 2 supervised home visits with a member of the field team, and a 1-day refresher session. CHWs were provided with a training handbook with case scenarios including what to do if the technology failed and underwent role plays for home visits. CHWs were trained to measure BP using the CRADLE VSA Blood Pressure Monitor (APEC) [32] and Hb using the handheld TrueHb Hemometer (Wrig Nanosystems Pvt Ltd) [38]. Guideline-based blood glucose testing for GDM involved a nonfasting OGTT delivered to the woman at home, followed by the CHW measuring a 2-hour capillary blood glucose level at the primary care subcenter [28]. Primary care doctors were also trained to use the SMARThealth Pregnancy mHealth platform and received individual refresher training on high-risk pregnancy conditions in a 2-hour session. The SMARThealth Pregnancy practices are outlined in Figure 4.

Intervention visits occurred at 3 strategic time points during and after pregnancy: an antenatal visit (28- to 36-week gestation) to detect high-risk cases prior to delivery and 2 postpartum visits, the first in week 1 to detect early postnatal complications and the second in week 6 to detect women with anemia or ongoing high BP or blood glucose who could then be linked to relevant government programs. Intervention visits were in addition to standard ANC and PNC.

All women were encouraged to attend the PHC after each home visit. Women requiring referral to secondary care were provided with a paper referral card by the primary care doctor and accompanied by the ASHA for visits to primary and/or secondary care.

### Control Group Practices

Control group participants received “enhanced” standard care, which included the awareness program in addition to routine ANC and PNC delivered by CHWs, as it was deemed ethically appropriate to provide the information on high-risk pregnancies and subsequent health to improve the overall well-being of pregnant women in the study districts.

The awareness program delivered to both intervention and control groups consisted of a 30-minute talk during the Village Health and Nutrition Days event and distribution of leaflets outlining the importance of attending ANC, the importance of identifying and treating anemia, HDP, and GDM in pregnancy and some of the long-term sequelae of these including their impact on the risk of future CMDs. An end-line data case report form was completed by a member of the research team for both the intervention and control groups.

### Study Outcomes and Analysis

The primary outcomes were to determine the feasibility and acceptability of the intervention and its implementation strategies, including recruitment of PHCs and participants, retention at 6-week postpartum, and fidelity to intervention practices. These data were used to understand patterns of recruitment and feasibility of conducting a larger cRCT. Secondary outcomes included clinical end points (BP and Hb): determining the prevalence of moderate to severe anemia (Hb <10.0 g/dL), HDP, and GDM within the study population. End of study BP and Hb clinical outcome data were collected primarily with the aim of assessing the feasibility of collecting these measures as potential clinical end points in a larger trial and to provide the CIs around these continuous variables for the intraclass correlation coefficient calculation ahead of a definitive trial. Descriptive statistics were used to reflect the above outcomes and compiled using SPSS (version 27; IBM Corp).

### Qualitative Substudy

To explore the acceptability of the intervention to CHWs and pregnant women, we conducted a qualitative substudy to explore the factors influencing the implementation and integration of the intervention into the daily work of CHWs. A purposive sample of pregnant or postpartum women and CHWs from the intervention group at each study site were approached to share their experiences of SMARThealth Pregnancy during the pilot study. Topic guides were developed for in-depth interviews (IDIs) and FGDs and piloted with the field team. SN conducted the IDIs and FGDs with a local research assistant, who provided translation support in the local languages. Discussions were audio recorded with consent, and transcripts were professionally transcribed into English. Data were analyzed using a framework analysis guided by normalization process theory (NPT), which addresses how interventions become embedded into the daily work of health care professionals [39].

### Effects of COVID-19 on Study

The pilot study started in October 2019, and India underwent a national lockdown for 2 months from March 24, 2020, because of the COVID-19 pandemic. This delayed recruitment at the control site in Andhra Pradesh and prevented the collection of end-line clinical outcome data in Haryana during April and May 2020. As screening pregnant women for high-risk conditions was deemed an essential service during the pandemic, ASHAs were permitted to conduct socially distanced home visits and telephone follow-up of postpartum women.

## Results

### Feasibility: Recruitment

All PHC leads approached to participate in the study agreed to be involved. A total of 4 primary care doctors, 10 ANMs, and 44 ASHAs at the intervention clusters participated. The time to recruit 50 pregnant women at each PHC cluster (N=200) ranged from 50 to 128 days (including a 60-day national lockdown), with a recruitment rate of 10 to 17 pregnant women (in their third trimester of pregnancy) per month. Recruitment was completed within 5 months across all 4 PHCs. All eligible women who were approached agreed to participate in the study.

### Retention and Postpartum Follow-Up

**Figure 5:**
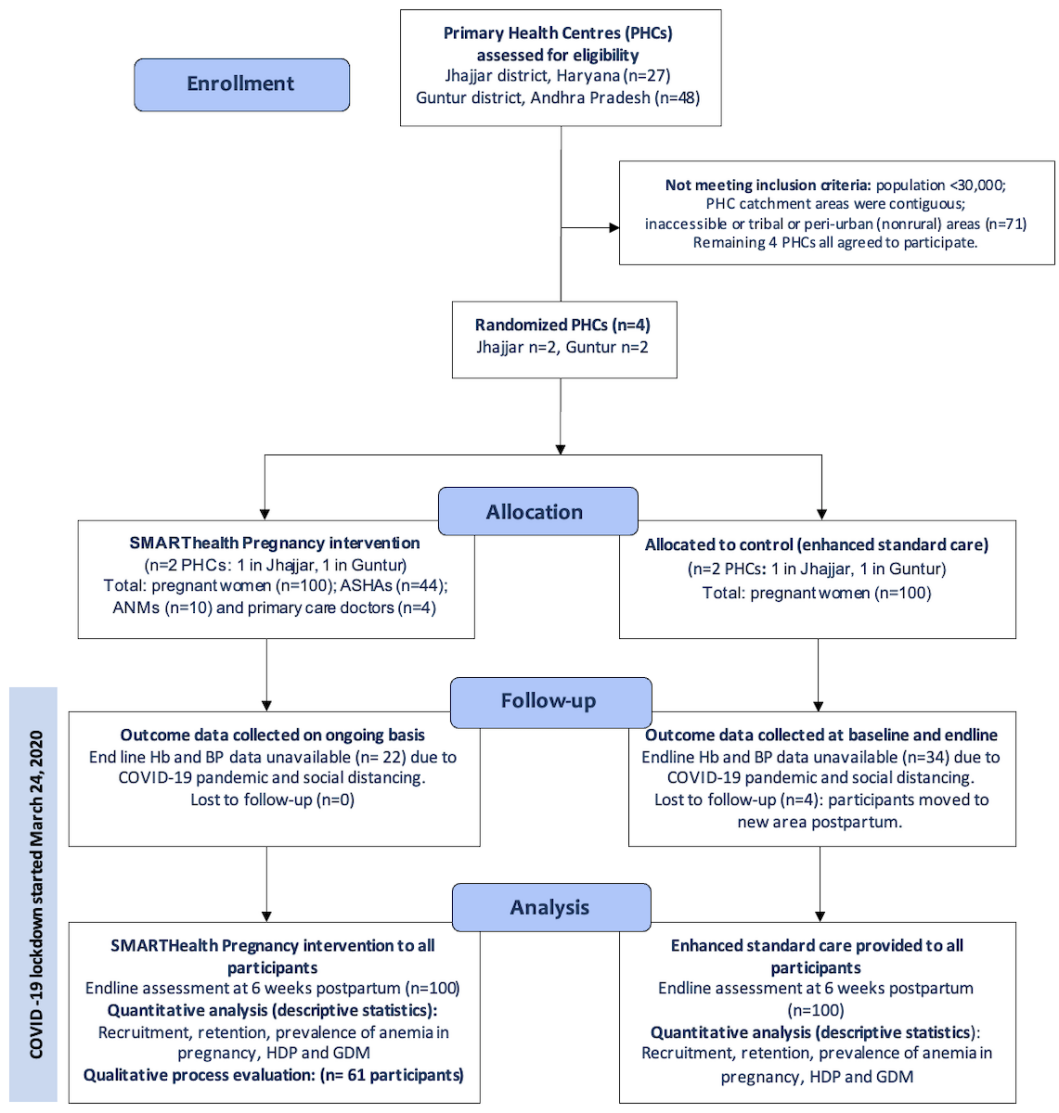
CONSORT flow diagram of study. ANM: Auxiliary Nurse Midwife; ASHA: Accredited Social Health Activist; BP: blood pressure; GDM: gestational diabetes mellitus; Hb: hemoglobin; HDP: hypertensive disorders of pregnancy; PHC: Primary Health Centre.

Four women (4/200, 2%) in the control group were lost to follow-up (2 at each study site). These 4 women moved out of the area to their mother’s village postpartum. There was no loss to follow-up in the intervention group (Figure 5). In Andhra Pradesh, 44% (22/50) of women in the intervention group saw a private obstetrician in the first 2 weeks postpartum, mostly (21/22) following a cesarean section. In Haryana, only 34% (17/50) of women saw a doctor in the first 2 weeks postpartum. No participant saw a doctor either privately or through government services from 2 weeks postpartum to the final 6-week postpartum visit. During this time, ASHAs were their only contact within the health system.

### Acceptability: Fidelity to Intervention Practices

Fidelity to intervention practices (in line with the study protocol) was evaluated through the completeness and timing of data entries into the mHealth platform. Prior to the global pandemic, ASHAs at both study sites completed all the first and second intervention visits per protocol (100% entries completed in full and on time). Due to COVID-19 social distancing restrictions, measurements of BP and Hb were not permitted at some study clusters during the pandemic. As a result, end-line BP and Hb clinical outcome data were not available for 22 of 100 participants in the intervention group and 34 of 100 participants in the control group. Where the end-line measurements were permitted, all ASHAs delivered the intervention in keeping with the study protocol (100% fidelity to intervention practices).

### Clinical End Points

Both intervention and control groups were comparable at baseline with regard to sociodemographic data (Table 1). Pregnant women participants on average had completed 12 years of schooling with comparable mean age, weight, and baseline Hb and BP.

**Table 1:** Baseline demographic data (n=100 for both the intervention and control groups) and end-line clinical data (n=78 for intervention group and n=62 for the control group).

			Intervention, mean (SD)		Control, mean (SD)		*P* value
**Baseline demographic data**							
	Age (years)	23.5 (3.5)		24.0 (3.6)		.32	
	BMI (kg/m^2^)	22.6 (4.0)		22.1 (4.1)		.38	
	Years of schooling	12.2 (3.7)		12.4 (4.0)		.71	
	Number of people in household	4.9 (2.1)		4.8 (1.9)		.72	
	Hb^a^ at antenatal booking visit (g/dL)	9.6 (1.4)		9.4 (1.4)		.31	
	Baseline study visit Hb (g/dL)	9.9 (1.7)		9.6 (1.7)		.21	
	Baseline systolic BP^b^	108 (11.3)		108 (12.4)		>.99	
	Baseline diastolic BP	70 (8.5)		70 (9.0)		>.99	
**End-line clinical outcome data**							
	End-line visit Hb (g/dL)	11.1 (1.7)		10.3 (1.7)		.01	
	End-line systolic BP	110 (11.6)		112 (13.4)		.35	
	End-line diastolic BP	72 (8.2)		73 (11.9)		.57	

^a^Hb: hemoglobin.

^b^BP: blood pressure.

### Prevalence of 3 Main High-Risk Conditions

The prevalence of moderate to severe anemia (Hb<10 g/dL) was high at both study sites. At baseline (third trimester of pregnancy): 47% (47/100) in the intervention group and 58% (58/100) in the control group. The study prevalence of HDP was 2.5% (5/200) and GDM was 2% (4/200).

### End-Line Clinical Data

At end line, the intervention group had a mean Hb (11.1, SD 1.7 g/dL) within normal range, in comparison with the mean Hb in the control group (10.3 SD 1.7 g/dL; indicative of postpartum anemia). BP values at end line were similar between groups, although this exploratory analysis did not adjust for clustering.

### Screening for GDM

At recruitment, 82% (163/200) of women had been screened for GDM before 28-week gestation; however, only 19% (37/200) reported having an OGTT (the gold standard test). Women who had not had an OGTT were offered one after recruitment, in line with government of India guidance [28]. All participants in the intervention group had an OGTT. Nevertheless, only 2% (4/200) of the study population were diagnosed with GDM. All 4 women diagnosed with GDM saw private obstetricians who did not reinforce the ASHAs’ advice for postpartum GDM follow-up as per government guidelines and were not offered further OGTT testing or ongoing medication by their health care providers, once their blood glucose levels had normalized after delivery.

### Qualitative Substudy: Acceptability of the mHealth Platform

A total of 61 participants, including CHWs (n=56) and pregnant or postpartum women (n=5), took part in 5 FGDs and 7 IDIs. Data were analyzed using NPT, which outlines 4 main constructs in relation to the implementation of an intervention. First, the sense-making work done by ASHAs and women to understand the value, impact, and importance of the intervention and a new set of practices (coherence); second, the relational work between ASHAs and the wider community to engage with and sustain the intervention practices (cognitive participation); third, the operational work CHWs undertook to implement the intervention (collective action); and finally, the reflective practices of CHWs in relation to the intervention that enabled it to become embedded in their daily work (reflexive monitoring).

CHWs were able to understand the value of the intervention practices in relation to previous ways of working (coherence). They felt that the intervention had improved their skills and through provision of mobile clinical decision support, the quality of the care they could deliver to women at home and had also improved their knowledge and confidence in their abilities (self-efficacy).

Previously ANMs used to do Hb or BP tests.... Now, since we are doing it, our knowledge improved. We did not know about this in-depth...we are very happy.

ASHA focus group, Andhra Pradesh

In particular, the intervention changed the nature of the relationships with other health professionals and pregnant women, with ASHAs gaining recognition of their skills from ANMs and primary care doctors, as well as heightened status within their communities (cognitive participation).

Now, we also know to measure BP, Hb.... Other people ask if the tests can be done to them too.... Now they want the tests to be done to them too!... Now we have some value in the village.... Now, people at least recognise us in our area.

ASHA focus group, Haryana

ASHAs particularly valued the mHealth platform, the traffic light system, and the visibility of clinical data to recall information and past medical history more easily. They reported that the mHealth platform was easy to use and had improved the quality of care they provided during home visits by providing ASHAs with the ability to give targeted advice to women at home during and after their pregnancy through mobile clinical decision support.

When we go back to that patient we are able to see the data which we had filled previously and at any point in time we can go back to the first visit and check and to the second visit and check. So it helps us to retain the data and to see it instantly.

ASHA, Haryana

ANMs also noticed an improvement in their skills and knowledge in relation to GDM.

We got to know [about GDM] only after you came.... We used to know about Hb and BP but we never used to ask about Diabetes.

ANM, Andhra Pradesh

The operationalization of community-based screening for GDM flattened the professional hierarchy between ANMs and ASHAs, who were required to work together to deliver OGTTs in the community (collective action). Both ANMs and ASHAs could see the value of working more as a team and appreciated acquiring the skills to deliver OGTTs in the community as part of their gold standard care for the rural population.

Previously...OGTT test was only done in private hospitals. Not all private hospitals had that too, madam! They used to conduct in only in few, selective, big corporate hospitals used to do it.... Tests like OGTT are very useful.

ANM focus group, Andhra Pradesh

ASHAs gained confidence (self-efficacy) in using the equipment, which was reflected in their greater knowledge of high-risk conditions in pregnancy, which in turn, influenced the pregnant women’s understanding of these conditions and improved the perceived quality of care received as part of government services.

I really like the ASHA worker because many things I don’t know about the pregnancy and I now know about these things because of ASHA workers.... It’s very nice...and these are done in a timely manner, and they have taken good care of me. They have taken care of us by doing blood tests....all are things very good.

Before pregnancy I didn’t know about the ASHA workers. After pregnancy I know them.... Seriously, the facilities here are very good. I appreciate all the things [they are doing] and it’s very good for me.

Postpartum woman, Haryana

ASHAs felt the SMARThealth Pregnancy intervention added value to home visits and could be integrated into their daily work without a perceived increase in workload.

It is a part of the work, but we get to know more information because of this.

ASHA, Haryana

During the study, ASHAs formed peer support groups to help each other in conducting home visits (reflexive monitoring). This led to reflective practice, problem-solving, and an ability to “learn” through using the mHealth platform.

ANM: They don’t ask us for any help on this.... They have good understanding. They discuss among 4-5 ASHA workers as a group, if there is any issue related to Tab or instrument. They are solving the problem by themselves.

ANM group, Andhra Pradesh

The SMARThealth Pregnancy was well received by all cadres of CHWs and by pregnant or postpartum women. It empowered ASHAs to provide care at home and counsel women. Pregnant women perceived the technology and level of care provided by ASHAs as high quality and this, in turn, elevated the status of ASHAs within their communities and among other health professionals. These factors led to the embedding of the intervention into their daily work and reflected the acceptability toward new ways of working.

## Discussion

### Principal Results and Comparison With Prior Work

Our study demonstrated that the SMARThealth Pregnancy intervention and implementation strategies were both feasible and acceptable to end users. Recruitment and retention of PHCs and participants to the pilot cRCT are feasible ahead of a larger clinical trial—recruitment being completed in under 5 months during the pandemic, with minimal loss to follow-up (4/200, 2%). Feasibility of the implementation strategies was reflected by high levels of fidelity and engagement with intervention practices. ASHAs and pregnant or postpartum women engaged strongly with the intervention (100% fidelity to intervention practices prepandemic) and expressed their ability to (1) learn about high-risk pregnancies from the mHealth platform, (2) share this learning with women within their communities (diffusion of knowledge), and (3) with each other, through peer support groups. The mHealth platform improved the perceived self-efficacy of ASHAs in relation to delivering home-based care to pregnant women, and both health care workers and women perceived an improvement in quality of perinatal care, skills (BP and Hb measurement), and professional recognition within their communities. The results suggest a possible improvement in mean Hb (anemia) in the intervention group; however, the significance of this needs to be interpreted with caution as the analysis did not adjust for clustering and would need to be confirmed in an adequately powered trial.

These findings reflect some of the methodological approaches adopted in the design and development of SMARThealth Pregnancy. First, sustained engagement with communities and health care workers at study sites through in-depth contextual work helped build trust between the research team and local communities. Early community engagement and stakeholder involvement are important for the timely and successful implementation of new interventions and the recruitment and retention of study participants [40-43]. The early contextual work at both study sites meant that the priorities of rural women and health care workers were integrated into the design of the intervention through human-centered approaches. As ASHAs and primary care doctors co-designed SMARThealth Pregnancy, they found it easy to navigate, implement, and integrate into their daily work, which was instrumental to the intervention’s acceptability.

Second, we adopted a theory-informed approach to intervention development, using the COM-B/behavior change wheel framework. The intervention addressed the barriers to behavior change of pregnant women and CHWs by improving their capabilities, opportunities, and motivations in relation to screening, referral, and counseling for high-risk conditions in pregnancy. Improved self-efficacy perceived by ASHAs in the qualitative study was a consequence of addressing their psychological and physical capabilities to perform BP and Hb testing at home and interpret readings through the mHealth platform. Furthermore, providing ASHAs with the physical and social opportunities (clinical decision support and BP/Hb devices) to conduct home visits improved their legitimacy and enhanced social and professional recognition. These factors are supported by the wider literature and impact CHW motivation [44] and performance [45].

The prevalence of moderate to severe anemia in pregnant women at baseline was high (47/100, 47% in intervention group and 58/100, 58% in control group). These findings are consistent with the wider literature [29,46-48]. Although clinical effectiveness was beyond the scope of this pilot study, we demonstrated a possible reduction in anemia (improved Hb) in the intervention group at end line. This finding may have been due to improvement in the knowledge of anemia among women and their households resulting from the ASHAs’ counseling during home visits, as highlighted in the qualitative substudy. The prevalence of HDP and GDM at our study sites (5/200, 2.5% and 4/200, 2%, respectively) was lower than that quoted in other studies [21,49-51]. This may reflect seasonal variations in BP or differing age and socioeconomic status [52-54] and arise from the variety of screening criteria and tests used to diagnose GDM in other studies [55-57]. Age-adjusted prevalence of GDM may be more in keeping with our findings [58], and the low prevalence we report may reflect the levels of poverty and rurality of our study population. Finally, embedding complex interventions into the daily work of health care professionals occurs as a result of people working individually and collectively to implement and integrate a new set of practices [39], requiring cooperation, buy-in, and ongoing commitment of health care workers and beneficiaries [59]. Health care workers shape how an intervention is delivered by the meaning they attribute to the intervention, which may differ across individuals, groups, and contexts [39,60]. NPT explained the factors contributing to the acceptability and embedding of SMARThealth Pregnancy into the daily work of ASHAs. ASHAs demonstrated understanding of the differences between previous ways of working, recognized the value of the mHealth platform, renegotiated their professional roles with ANMs and doctors, and adopted a team-based approach to health care delivery with regards to GDM screening as a result of the intervention. Furthermore, the qualitative substudy highlighted that ASHAs did not see SMARThealth Pregnancy as a burden in terms of workload, which contributed to its perceived value.

Our study was conducted during the COVID-19 pandemic. During this time, ASHAs were the only health service contact for pregnant and postpartum women in our study sites. Our study demonstrated that ASHAs are well placed to deliver postpartum interventions and offer health-related counseling to high-risk pregnant rural women at home when provided with adequate support. Our model of task sharing to deliver home-based point-of-care testing for anemia and BP was seen as very valuable to women and the community in our study. Task sharing has been identified as a method of meeting workforce shortages in rural areas [61-63]. We have demonstrated that providing ASHAs with the tools to engage in effective task sharing has the potential to improve perceived quality of care, improve self-efficacy of CHWs, and encourage learning for both CHWs and patients.

### Strengths and Limitations

We have presented a robust and innovative approach to theory-informed intervention development and evaluation to address the immediate and long-term needs of high-risk pregnant women in relation to 3 priority areas. Our findings have informed the need for a larger trial. Limitations of our study relate to disruptions in study procedures as a result of the 2020 pandemic, which limited our end-line clinical outcome data collection. Any significant differences in end-line clinical data would need to be confirmed through an adequately powered trial, adjusting for clustering, which was beyond the scope of this pilot study. We further envisaged that women would visit their primary care doctor after each study visit to strengthen the links with government services and the PHC; however, in practice, women bypassed primary care and sought obstetricians in secondary care directly, particularly in the postpartum period, when there are cultural taboos related to women leaving their homes for 40 days. Due to the fragmented nature of PNC, women received inconsistent advice, particularly relating to future risks of CMDs for women with GDM. This highlighted the importance of whole-systems approaches to education and training of health care workers to advise high-risk pregnant women, and the need to involve secondary care providers in community-level interventions to reinforce evidence-based follow-up of high-risk pregnant women beyond the immediate postpartum period.

### Conclusions

SMARThealth Pregnancy is a feasible and acceptable multifaceted complex intervention to support CHWs to deliver home-based care for high-risk pregnant women in the context of 2 diverse states in rural India. The intervention resulted in a model of task sharing for the integrated ANC, PNC, and ongoing care of women. Our successful pilot study has informed the decision to proceed with a definitive trial of clinical effectiveness using the SMARThealth Pregnancy approach to integrate pregnancy and NCD care. The goal is an effective, affordable, and acceptable model of integrated care that uses pregnancy as an opportunity to improve women’s lifelong health.

## Appendix

Multimedia Appendix 1 CONSORT-eHEALTH checklist (V 1.6.1).

## Abbreviations

ANC: antenatal care
ANM: auxiliary nurse midwife
ASHA: accredited social health activist
BP: blood pressure
CHW: community health worker
CMD: cardiometabolic disorder
cRCT: cluster randomized controlled trial
CVD: cardiovascular disease
FGD: focus group discussion
GDM: gestational diabetes mellitus
HDP: hypertensive disorders of pregnancy
IDI: in-depth interview
mHealth: mobile health
NCD: noncommunicable disease
NPT: normalization process theory
OGTT: oral glucose tolerance test
PHC: primary health center
PNC: postnatal care

## Data Availability

The data sets generated and analyzed during this study are not publicly available due to restricted permissions within the districts involved in the study but are available from the corresponding author upon reasonable request.
